# Combining Different Wearable Devices to Assess Gait Speed in Real-World Settings

**DOI:** 10.3390/s24103205

**Published:** 2024-05-17

**Authors:** Michele Zanoletti, Pasquale Bufano, Francesco Bossi, Francesco Di Rienzo, Carlotta Marinai, Gianluca Rho, Carlo Vallati, Nicola Carbonaro, Alberto Greco, Marco Laurino, Alessandro Tognetti

**Affiliations:** 1 National Research Council, Institute of Clinical Physiology, 56124 Pisa, Italy; pasquale.bufano@phd.unipi.it (P.B.); marco.laurino@cnr.it (M.L.); 2 Department Information Engineering, University of Pisa, 56122 Pisa, Italy; francesco.bossi@ing.unipi.it (F.B.); francesco.dirienzo@phd.unipi.it (F.D.R.); carlotta.marinai@phd.unipi.it (C.M.); gianluca.rho@phd.unipi.it (G.R.); carlo.vallati@unipi.it (C.V.); nicola.carbonaro@unipi.it (N.C.); alberto.greco@unipi.it (A.G.); alessandro.tognetti@unipi.it (A.T.); 3 Department of Surgical, Medical and Molecular Pathology and Critical Care Medicine, University of Pisa, 56126 Pisa, Italy

**Keywords:** daily life monitoring, gait speed estimation, machine learning, mobility analysis, smart sensors, smartphone, smartwatch, smart shoes, telemedicine, wearable devices

## Abstract

Assessing mobility in daily life can provide significant insights into several clinical conditions, such as Chronic Obstructive Pulmonary Disease (COPD). In this paper, we present a comprehensive analysis of wearable devices’ performance in gait speed estimation and explore optimal device combinations for everyday use. Using data collected from smartphones, smartwatches, and smart shoes, we evaluated the individual capabilities of each device and explored their synergistic effects when combined, thereby accommodating the preferences and possibilities of individuals for wearing different types of devices. Our study involved 20 healthy subjects performing a modified Six-Minute Walking Test (6MWT) under various conditions. The results revealed only little performance differences among devices, with the combination of smartwatches and smart shoes exhibiting superior estimation accuracy. Particularly, smartwatches captured additional health-related information and demonstrated enhanced accuracy when paired with other devices. Surprisingly, wearing all devices concurrently did not yield optimal results, suggesting a potential redundancy in feature extraction. Feature importance analysis highlighted key variables contributing to gait speed estimation, providing valuable insights for model refinement.

## 1. Introduction

The assessment of mobility loss in real-world settings and in the long term could have a profound effect on medical practice, much like how cardiac Holter monitoring revolutionized the evaluation of cardiac pathologies in the past. Indeed, the decline in mobility is a morbidity factor in various pathophysiological conditions, including heart failure, Chronic Obstructive Pulmonary Disease (COPD), and neurodegenerative diseases. In the medical field, mobility analysis holds significance for various purposes. For example, in patients with Parkinson’s disease, both to clarify gait abnormalities [1] and to investigate the effects of different therapies [2,3], in mental disorders, where mobility alterations are a sign of disease (depression, schizophrenia, anxiety disorders, etc.) to predict relapses or exacerbations [4], and in various chronic lung conditions to assess disease severity [5,6]. In particular, walking speed is a valid, reliable, and sensitive measure for assessing health in a wide range of populations [7]. The gait speed (GS) has been identified as the “sixth vital sign” [8]; it is a simple measure for predicting health status with very important clinical applications for a wide range of pathological conditions [9].

Over past years, the estimation of gait speed has been conducted through several validation studies using various tools (e.g., wrist sensors [10], lower-back-worn inertial sensors [11], smartphone sensors [12], smartwatch sensors [13], or a combination of three accelerometers, on the thigh, on the sacrum, and on the shanks [14]). Despite the significance of previous research, real-world mobility assessment has not yet been integrated into current clinical practices.

The aim of this work is to propose a new method for estimating gait speed (GS) from a heterogeneous set of wearable devices, which can be employed one by one, collectively, or in any combination. For each combination of wearable devices, we assessed the estimation accuracy and explained the features that contribute most significantly to the estimation. To increase the generalization of our approach, we combined two common commercial wearable devices (a smartphone and a smartwatch) with a gait-analysis-dedicated wearable system (a smart shoe integrating inertial and pressure sensors). These devices can capture gait dynamic information through different modalities. Our focus is on describing the device’s performance and identifying optimal combinations that individuals may utilize in their daily lives.

This work is part of the European Union-funded TOLIFE project [15], in which the focus is on COPD. The goal is to collect data from the daily lives of COPD patients using non-invasive smart sensors to be used for the development and clinical validation of an artificial intelligence (AI) solution to optimize and personalize treatment and improve the quality of life of COPD patients [16]. Indeed, a recent systematic review and meta-analyses [17] have shown several associations between mobility parameters detected by non-invasive sensor devices and COPD outcomes, such that a loss of mobility is associated with an increased mortality risk. Moreover, slow GS is associated with increased healthcare utilization. Since these results demonstrate that a loss of mobility is one of the most important prognostic factors for COPD outcomes, studying the mobility of COPD patients is pivotal to the process of acquiring daily life parameters to be used for the creation of predictive algorithms underlying AI.

To estimate gait speed, we built a multiple linear regression model for each of the seven possible combinations of devices (i.e., phone, watch, shoes, “phone + watch”, “phone + shoes”, “watch + shoes”, and “all devices”). To train and assess our model, we used data collected in a protocol in which sensor data from the three wearable devices were associated with data collected by a reference system during controlled walking tasks. For the experimental phase, we enrolled 20 healthy subjects, each wearing a set of wearable devices (smartphone, smartwatch, and smart shoes) and the reference system used to obtain the gait speed. The reference system was the Awinda inertial motion tracker coupled with the MVN Analyse software (version 2023.0), both supplied by Xsens [18] (Enschede, Netherlands). We asked each subject to perform a modified version of the Six-Minute Walking Test (6MWT) [19] three times at three different paces, medium, slow, and fast, in order to cover a wider range of speeds.

The results obtained showed a reasonably accurate performance in estimating walking speed with root mean square errors consistent with the relevant literature studies [10,11,12,14], which, however, focused on methods applied to single device combinations. To the best of our knowledge, this is the first time that the impact of using any combination of three wearable devices to assess walking speed has been quantified, aiming to provide more ecological real-world monitoring of health conditions. Indeed, our method automatically selects the best feature sets given the combination of devices, and it is always able to provide the best estimation. By applying this approach to the daily life condition of COPD patients, the use of multiple data sources allows us to obtain data throughout the day without interruption, as these can be used simultaneously (a robust measurement to obtain better performance from the estimation algorithms) or separately (when, for example, one device runs out of power or stops working). Furthermore, they allow the patient to have greater compliance since having a set of three different devices enables them to choose the most suitable device for the daily activity he/she wishes to carry out.

The organization of this paper is as follows. In Section 2, we introduce the experimental acquisition protocol and the devices under investigation. We also illustrate how the data were processed and the approach adopted for the implementation and validation of the machine learning models. In Section 3, we display the results obtained by reporting the performance of the algorithms under different conditions. Finally, in Section 4, we discuss the results to conclude in Section 5.

## 2. Materials and Methods

### 2.1. Instrumentation

#### 2.1.1. Wearable Devices

**Figure 1:**
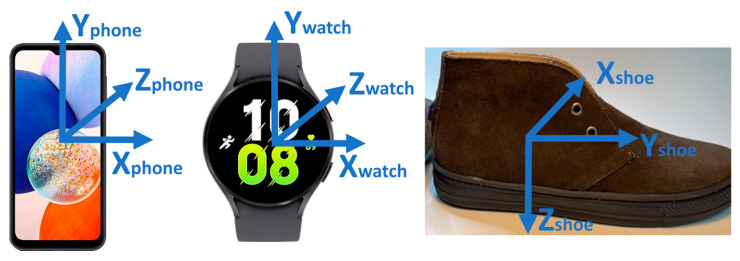
Smartphone (**left**), smartwatch (**center**), and smart shoe (**right**). Local reference frames associated with the inertial sensors are reported.

**Figure 2:**
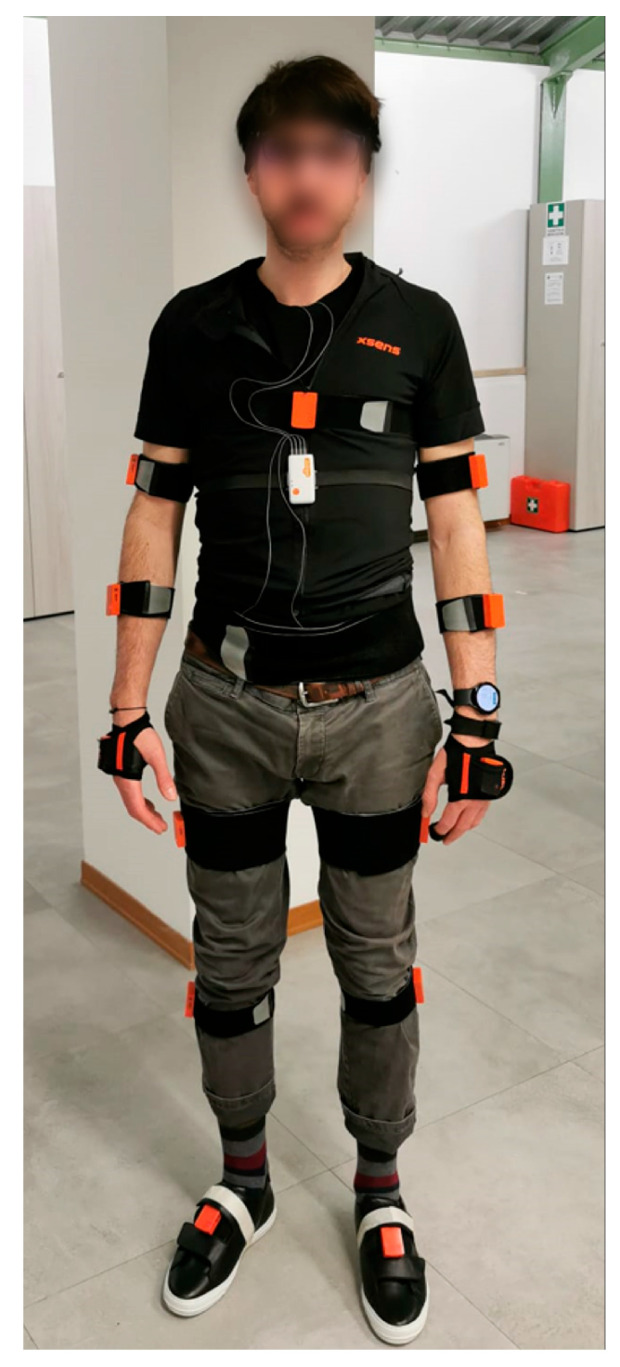
Subject wearing the reference system and the wearable devices.

In this study, we used the wearable device set of the TOLIFE sensor platform, specifically developed for the home monitoring of COPD patients [15,20]. The device set was selected to fulfill the needs established by the EU-funded TOLIFE project, which requires the collection of raw data related to modulating factors, performance, and symptoms of patients with Chronic Obstructive Pulmonary Disease (COPD). These data are necessary to construct algorithms based on artificial intelligence for (1) the early detection of COPD exacerbations and (2) the estimation of the evolution of health-related quality of life, functional exercise capacity, and dyspnea in COPD patients. The TOLIFE sensor set employs non-invasive wearable and non-wearable sensors, consisting of the following sub-devices: (1) a smart mattress cover and bedroom box case, (2) smart shoes, (3) a smartphone, (4) a smartwatch, and (5) a spirometer. The devices used in this study comprise the wearable set of TOLIFE devices and will be specifically employed to extract the mobility parameters of COPD patients. The set comprises a smartphone (the Samsung Galaxy A14, Figure 1 left), a smartwatch (the Samsung Galaxy Watch 5, Figure 1 center), and a pair of smart shoes (Figure 1 right). From both the smartphone and smartwatch, we collected inertial sensor data (accelerometer and gyroscopes for the smartwatch; accelerometers and orientation for the smartphone). Smart shoes are a research prototype developed as an integrated solution to record and transmit gait analysis information to mobile phones. They are adapted from the prototypes described in our previous research [21,22] and are specifically tailored to the TOLIFE project [15]. The smart shoes are battery-powered and have an electronic unit integrated into the heel region of the insole. The electronic unit includes a digital inertial measurement unit (3-axis accelerometer and gyroscope, LSM6DSL by STMicroelectronics) and a Bluetooth low-energy connection. In addition, the smart shoes have three pressure sensors integrated under the insole (two in the forefoot and one in the heel region) to monitor the mechanical interaction of the foot with the ground (FSR 402 by Interlink). We developed two custom Android applications to collect data from the smartphone and smartwatch sensors. The smartphone application was also used to collect data from the smart shoes. Table 1 reports the acquired signals for these devices and their respective sampling frequencies. Figure 2 shows the reference system and wearable devices on a subject. The reference system includes the inertial units of the AWINDA motion tracker (orange units), the smart shoes, the smartwatch on the wrist, and the smartphone in the front pocket.

#### 2.1.2. Reference System

To obtain a reference measurement for the GS, we employed the AWINDA inertial motion tracker coupled with the MVN Analyze software, both provided by Xsens [23]. The Xsens Awinda system is composed of 17 wireless inertial measurement units. The MVN Analyze software provides tools for visualizing and interpreting movement data captured by Xsens sensors, including the Center of Mass (CoM) velocity. We used the horizontal component of the CoM velocity extracted by MVN Analyze as a reference for gait speed.

### 2.2. Experimental Protocol

This study was composed of twenty healthy subjects, 11 females and 9 males, aged 27.6 ± 1.6 years. Each subject wore the reference system and the wearable devices, with the smartwatch on the left arm and the smartphone placed in the trouser’s front pocket. Each participant was asked to perform a modified version of the Six-Minute-Walking-Test (6MWT), a widely recognized and standardized assessment tool to assess functional autonomy, especially in subjects with compromised lung function [19]. The original clinical version of the test consists of measuring the distance walked by a person in six minutes along a 30 m flat path, walking as fast as possible [24]. Our modified version took place along a 10 m flat path, with a turning radius of about 50 cm available to make directional changes. Each participant was asked to perform the test three times at three different paces, medium, slow, and fast, to cover a wider range of speeds. They were encouraged to self-select the pace during each trial. While performing the test, the devices were always worn in the same position. In particular, the watch was placed on the left wrist, and the phone was in the left pocket, with the screen facing the thigh and the *Y*-axis facing upward.

### 2.3. Algorithm for Gait Speed Estimation

As previously introduced, we aimed to build and assess a gait speed estimation algorithm capable of providing the best estimation for each possible wearable device combination. Therefore, we trained seven machine learning models on the following combinations of wearable devices: (i) phone, (ii) watch, (iii) shoes, (iv) phone + watch, (v) phone + shoes, (vi) watch + shoes, or (vii) all the devices.

The model chosen for each combination-specific GS estimation algorithm was a multiple linear regression with 11 terms (intercept and 10 regressors) as follows:

(1) $$\hat{y}={\beta_{0}+\beta }_{1}*f_{1}+...+\beta_{10}*f_{10}$$

where $\hat{y}$ is the estimated GS, $\beta_{j}$ are the regression coefficients, and $f_{j}$ are the features. Note that the features ($f_{j}$) vary across the different models according to the feature selection stage described later in this section.

**Figure 3:**
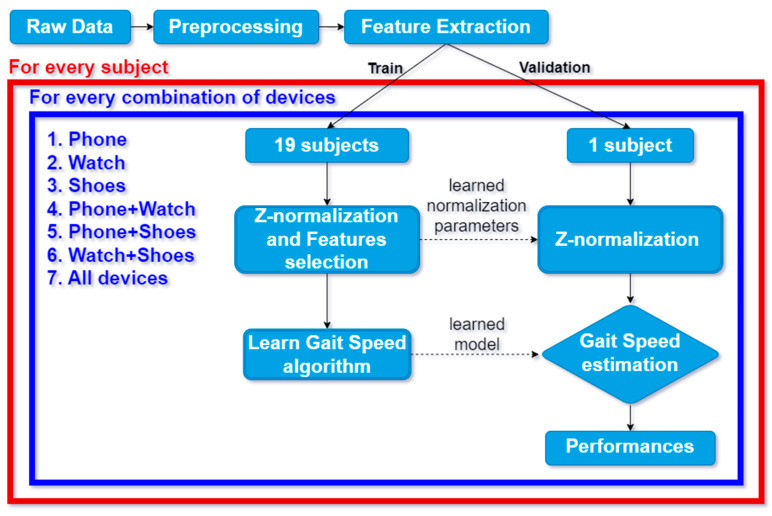
Workflow for gait speed estimation. The blocks within the blue box are repeated for every combination of devices. Meanwhile, the blocks inside the red box, which encompassed the blue box as well, are carried out for each subject in the cross-validation process.

The workflow reported in Figure 3 describes the steps needed to train the machine learning-based models capable of estimating walking speed from the data collected by different combinations of wearable devices. After pre-processing the raw signals of the wearable devices, a set of features was extracted and selected. These features were used to train a linear regressor for each of the seven possible combinations of wearable devices. The operations described in Figure 3 (i.e., feature selection, z-normalization, and GS estimation model training and validating) were carried out, validating one subject at a time, thus performing a leave-one-subject-out cross-validation to separately evaluate the performance of the models.

In the first stage of the proposed workflow, we preprocessed the raw sensor data to derive two datasets. To preserve information about gravity direction from the accelerometer signals, the first dataset included low-frequency components, which were obtained by filtering the signals with a low-pass filter (ft = 10 Hz). The second dataset excluded low-frequency components and was obtained by using a band-pass filter (ft1 = 0.1 Hz, ft2 = 10 Hz). Afterward, we extracted features from this dataset to train the models.

To extract the features, all signals were first segmented, adopting 5 s sliding windows with 80% overlap. For every derived time window, the following features were extracted for each of the components of the acquired signals and their modulus: (i) Mean, (ii) STD (Standard Deviation), (iii) CV (Coefficient of Variation), (iv) RMS (Root Mean Square), (v) Range, (vi) Max value, (vii) MCR (Mean Crossing Rate), (viii) PF (Peak Frequency), (ix) SMA (Signal Magnitude Area, computed once per sensor), and (x) Shannon Entropy. Therefore, for every sensor, we obtained 4 values for each of the features, except for the SMA, from which we obtained only 1. Combining the two datasets, we obtained 74 features for each sensor. The phone and watch, which acquire signals from accelerometers and gyroscopes, have 148 features, while shoes, which also acquire data from pressure sensors, have 222 features. Furthermore, the obtained features were z-normalized to prevent the estimation from being biased by different scales and to obtain comparable coefficients, which will improve the explainability of the model.

The features used to train the model were selected independently for every device. The adopted criterion to add terms was the Bayesian information criterion (BIC). The scoring was based on the likelihood function but also considered the complexity of the model, introducing a penalty for the number of parameters of the model. The model admitted only an intercept term and linear terms for each predictor, improving the explainability of the model. Every model was built starting with the intercept, and the stepwise was free to add terms based on the BIC criterion. For each model, we set a maximum of 10 terms; stepwise, we never stopped at a lower number of features, obtaining Equation (1). We also investigated the performances by varying the size of the time windows. Windows of 2.5, 5, 10, and 20 s were tested. We chose the overlapping between the windows to always ensure 1 sample per second.

### 2.4. Algorithm Evaluation

#### 2.4.1. Error Metrics

To evaluate the agreement between the GS measured by the reference system and the estimated one, we utilized the Bland–Altman and correlation plots, reporting the limits of agreement, the bias, and the coefficient of determination (R2). We performed the Bland–Altman and correlation plots also for the six-minute walking distance (6MWD), obtained by GS estimation by using the following formula:

(2) $$\hat{6MWD}=\left(\frac{1}{n}\sum_{i=1}^{n}{\hat{y}}_{i}\right)*360s$$

where ${\hat{y}}_{i}$ is the estimated GS for every time window and *n* is the number of the time windows extracted from the 6MWT under consideration.

We calculated the root mean square error (RMSE) of the GS estimation as follows:

(3) $$RMSE=\sqrt{\frac{1}{n}\sum_{i=1}^{n}{\left({\hat{y}}_{i}-y_{i}\right)}^{2}}$$

where $y_{i}$ is the speed provided by the Xsens and ${\hat{y}}_{i}$ is the speed estimated by the regression algorithm.

We then calculated the relative percentage error (ε_r_) on the 6MWD estimation as follows:

(4) $$\varepsilon_{r}=\left|\frac{6MWD-\hat{6MWD}}{6MWD}\right|*100$$

#### 2.4.2. Feature Interpretability

We performed the analysis described in this section to identify the most important features of each combination of devices. Each β coefficient of the regression was linearly associated with one predictor. Due to the z-normalization, the coefficients will be directly comparable.

To find the most important features, we investigated the training data following these steps:

1. Selection of significant features: we select features found to have been chosen in at least one model and found to be significant (*p*-value < 0.001);
2. First drop-out stage: for each combination, we drop out the features selected in less than half of the folds of the validation, i.e., 10 folds;
3. Second drop-out stage: through observing that each device can appear in 4 of the 7 combinations, we will select, for each device, those that appear in more than half of the possible combinations, i.e., 2 combinations.

The dataset is available on Zenodo (https://doi.org/10.5281/zenodo.11091279, accessed on 29 April 2024).

## 3. Results

### 3.1. Error Metrics

Table 2 reports the RMSE and Bland–Altman’s indices (limits of agreement, bias, and R2) for GS estimation obtained using features from different device combinations. The first column of Table 2 displays the RMSE for GS across different combinations of devices. The subsequent columns show the lower and the upper limits of agreement of the Bland–Altman analysis, along with the bias. The last column reports the coefficient of determination derived from the correlation plot.

As shown in Table 2, the best performance in terms of RMSE is achieved for the combination “Watch + Shoes”. On the other hand, the worst performance is obtained when the “Shoes” device is used alone.

Figure 4 and Figure 5 show the correlation and the Bland–Altman plots of the GS estimation for the configuration with the best RMSE (i.e., “Watch + Shoes”). These plots aggregate the estimations of all 20 subjects in the cross-validation. In Appendix A, in Figure A1, Figure A2, Figure A3, Figure A4, Figure A5, Figure A6, Figure A7, Figure A8, Figure A9, Figure A10, Figure A11 and Figure A12, we report the correlation and Bland–Altman plots for the combinations “Phone”, “Watch”, “Shoes”, “Phone + Watch”, “Phone + Shoes”, and “All Devices”.

The first column of Table 3 reports the ε_r_ for the 6MWD estimation. The subsequent columns show the lower and the upper Bland–Altman’s limits of agreement and the bias. The last one reports the coefficient of determination of the correlation plot.

Considering the percentage error, the best performance for GS estimation was obtained from the combination “Watch + Shoes”, while the worst performance was obtained from the “Shoes” device used alone.

**Figure 6:**
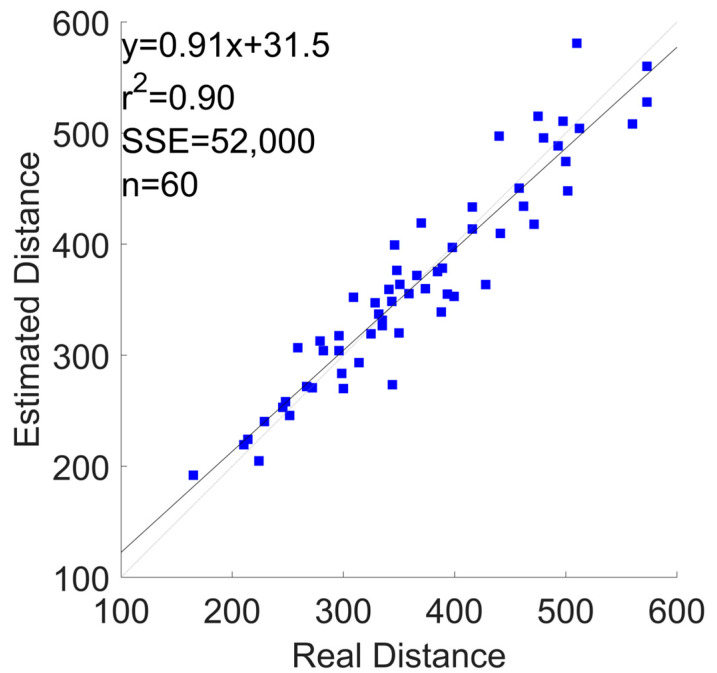
6MWD estimation using a correlation plot for the “Watch + Shoes” combination.

**Figure 7:**
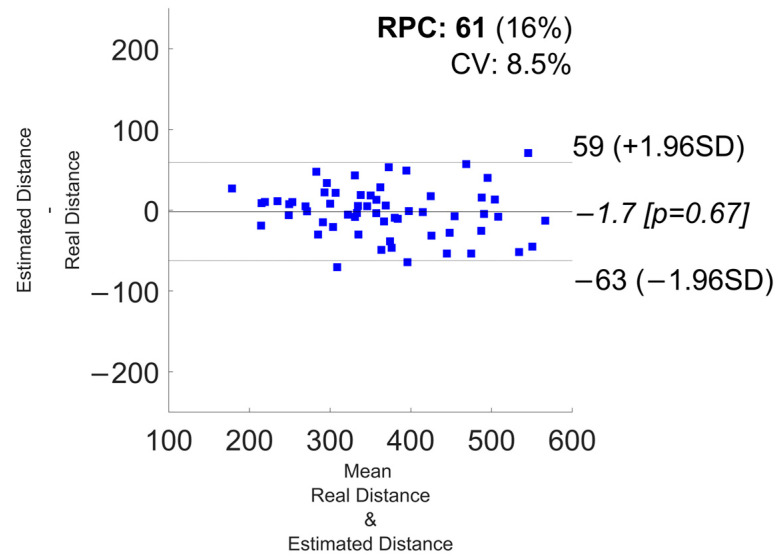
6MWD estimation using a Bland-Altman plot for the “Watch + Shoes” combination.

**Figure A13:**
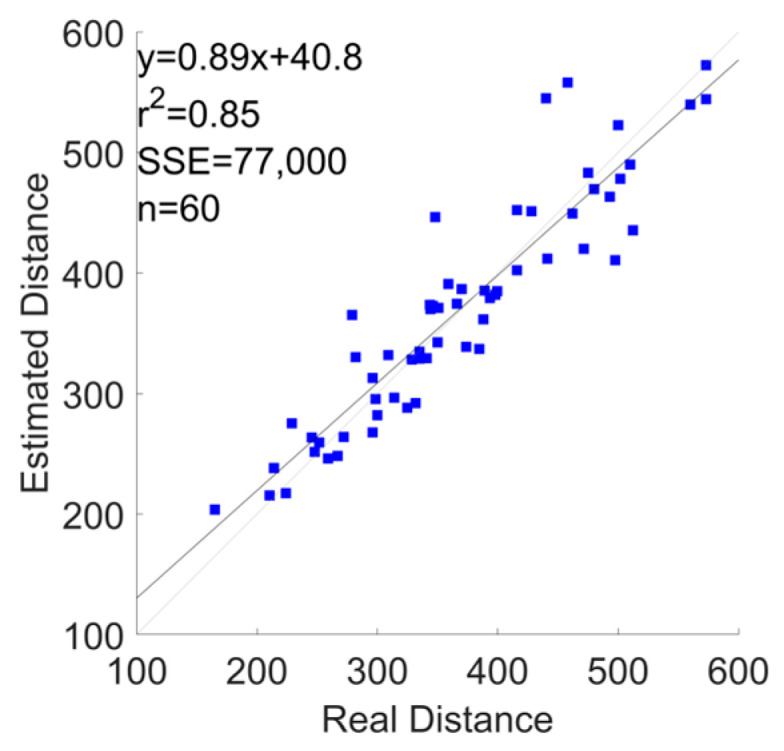
GS estimation using a correlation plot for “Phone” configuration.

**Figure A14:**
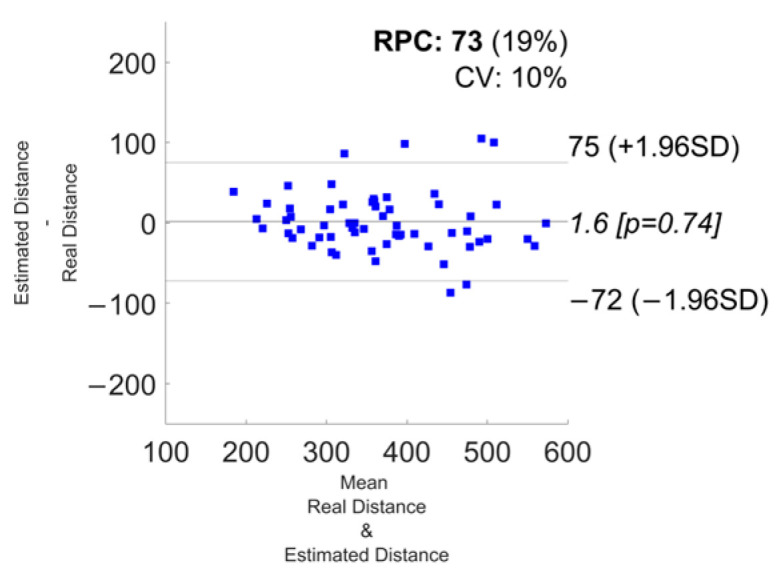
GS estimation using a Bland–Altman plot for “Phone” configuration.

**Figure A15:**
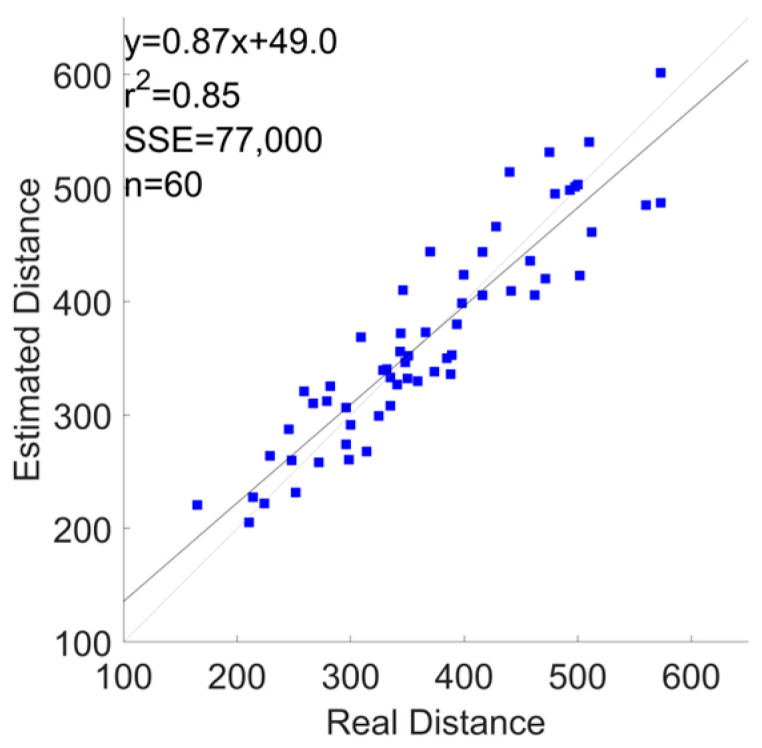
GS estimation using a correlation plot for “Watch” configuration.

**Figure A16:**
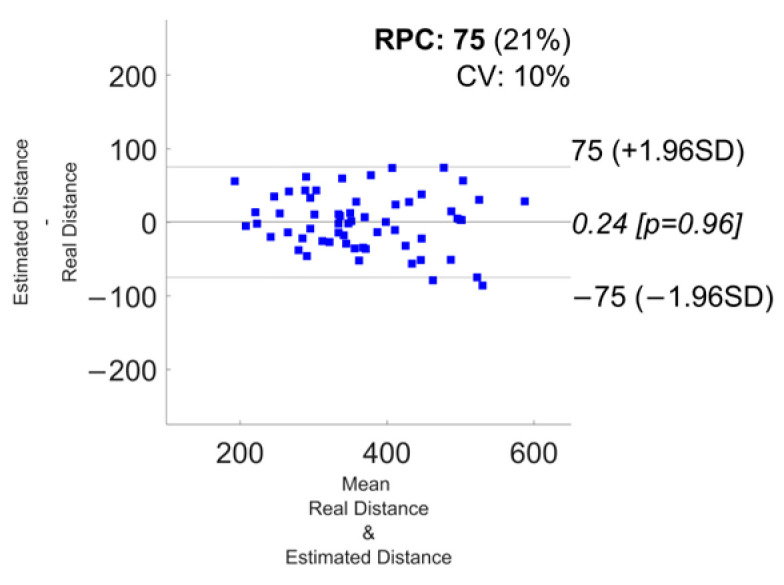
GS estimation using a Bland–Altman plot for “Watch” configuration.

**Figure A17:**
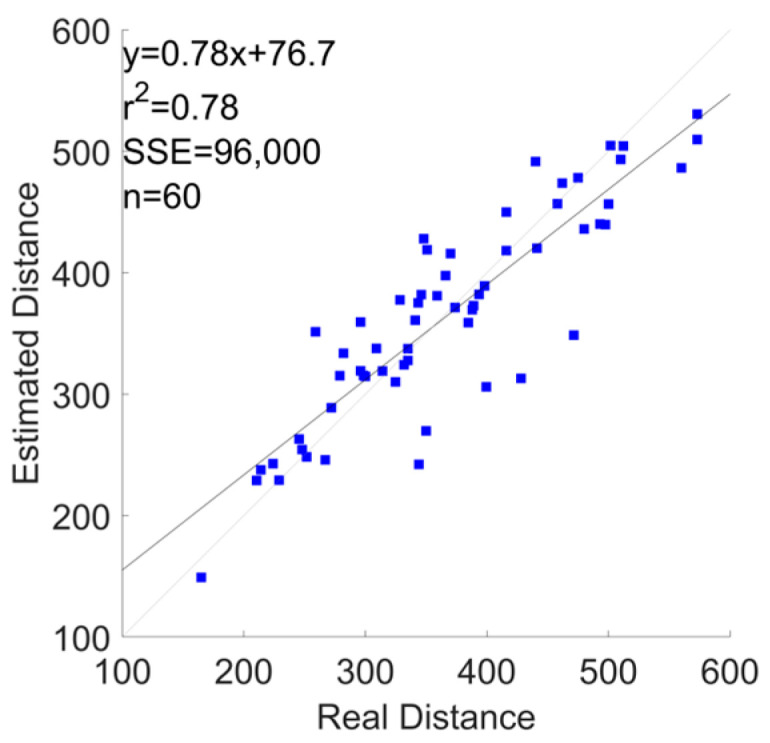
GS estimation using a correlation plot for “Shoes” configuration.

**Figure A18:**
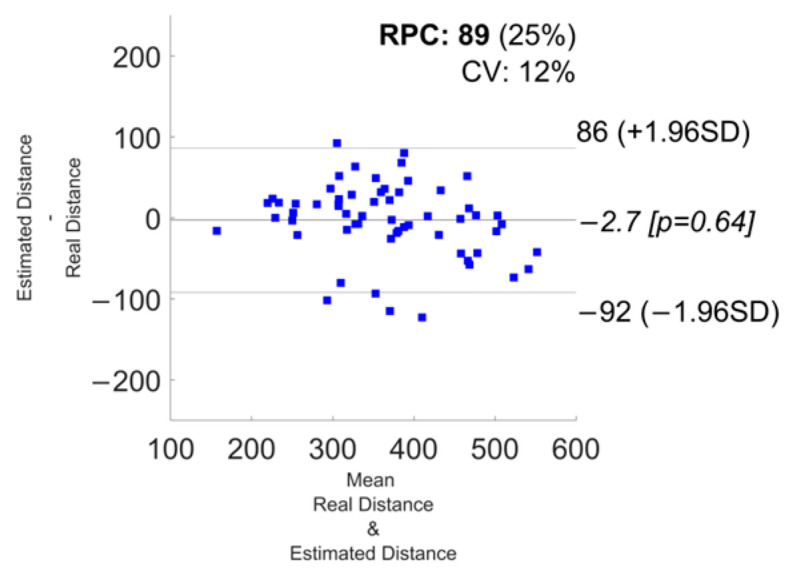
GS estimation using a Bland–Altman plot for “Shoes” configuration.

**Figure A19:**
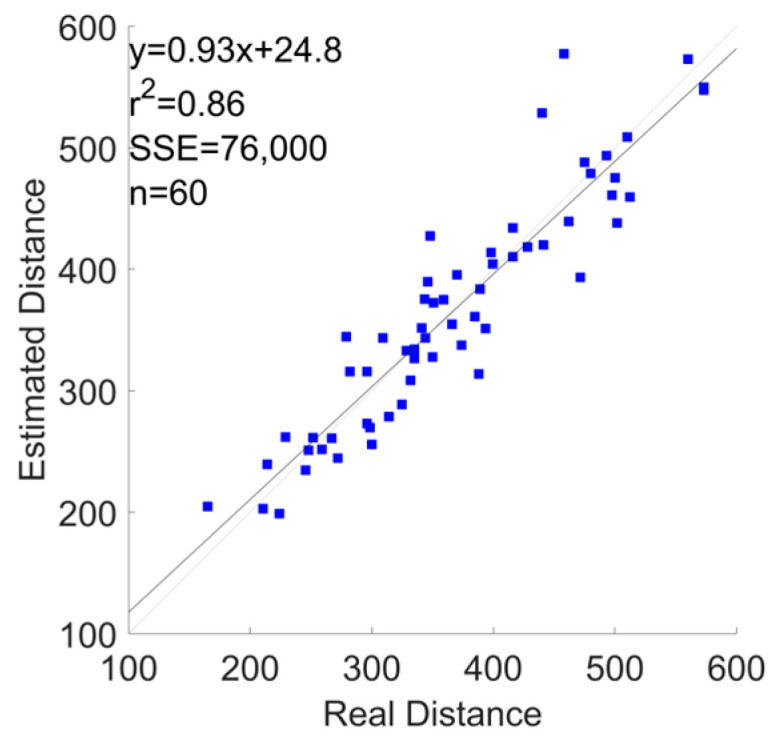
GS estimation using a correlation plot for the “Phone + Watch” configuration.

**Figure A20:**
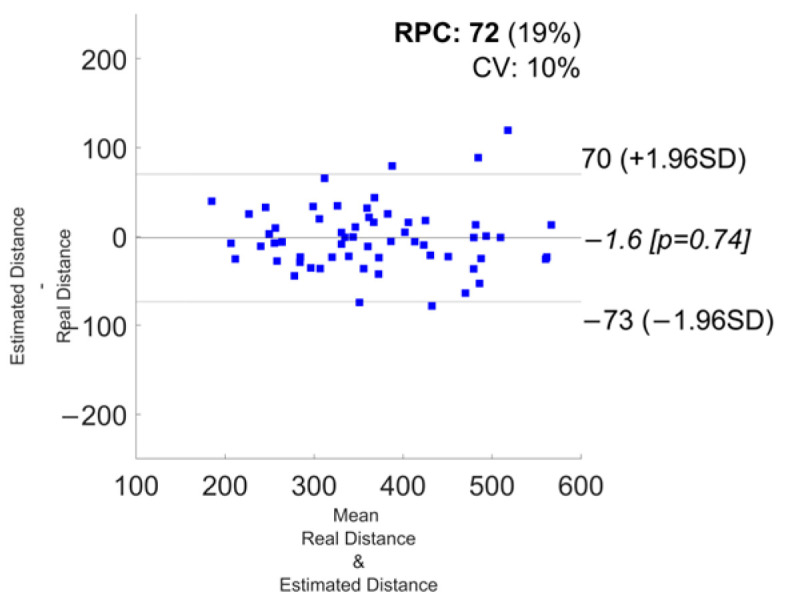
GS estimation using a Bland–Altman plot for “Phone + Watch” configuration.

**Figure A21:**
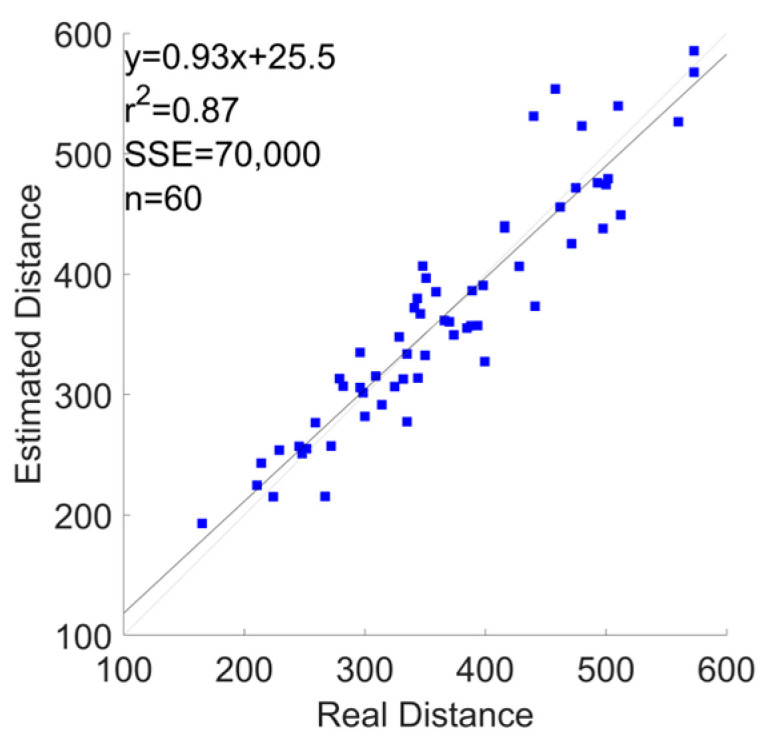
GS estimation using a correlation plot for the “Phone + Shoes” configuration.

**Figure A22:**
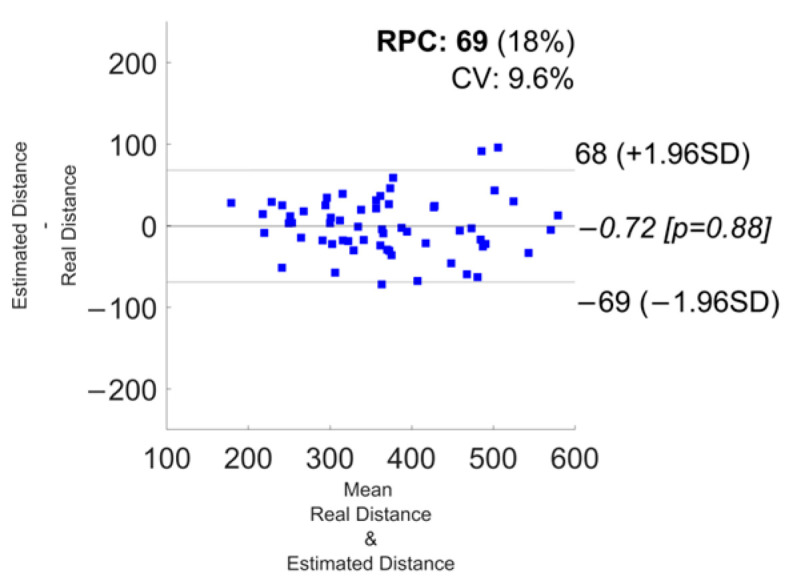
GS estimation using a Bland–Altman plot for “Phone + Shoes” configuration.

**Figure A23:**
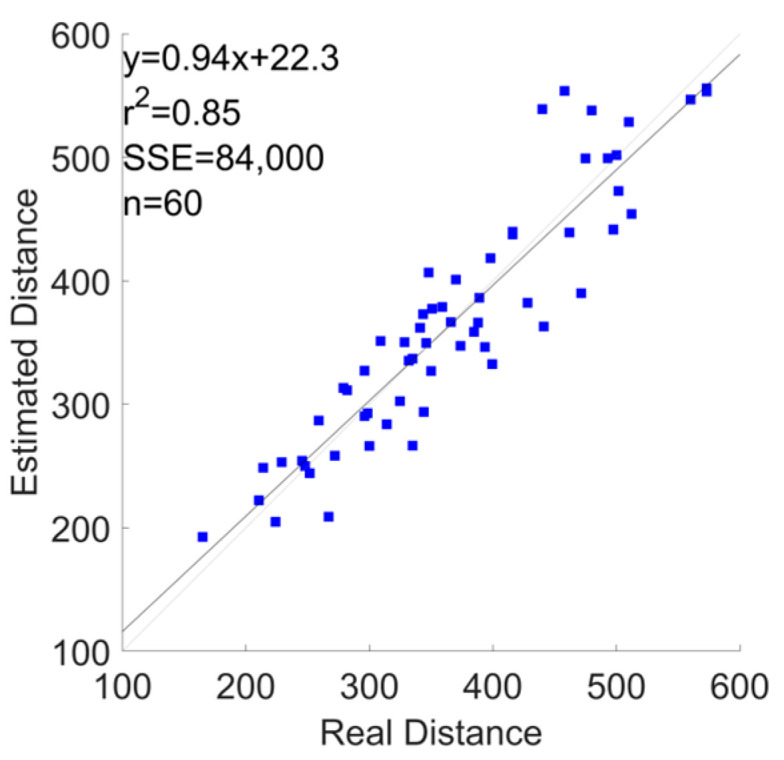
GS estimation using a correlation plot for “All Devices” configuration.

**Figure A24:**
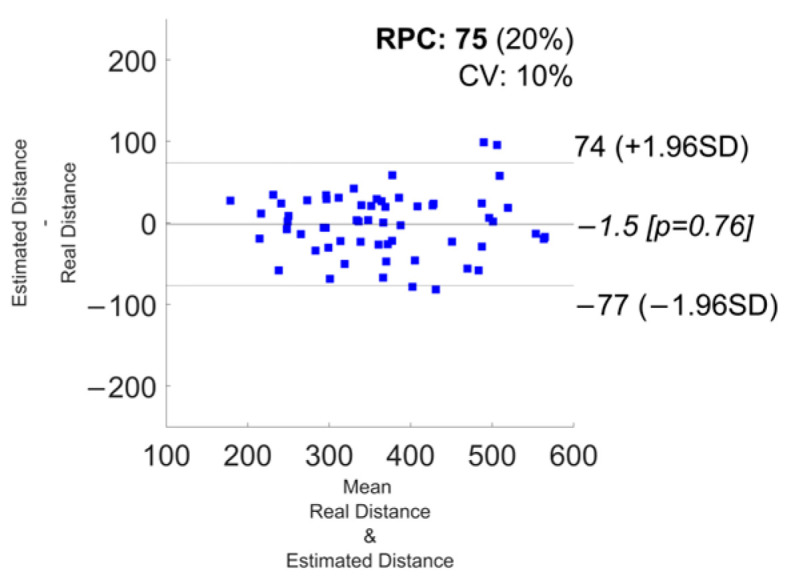
GS estimation using a Bland–Altman plot for “All Devices” configuration.

Figure 6 and Figure 7 show the correlation and the Bland–Altman plots of the 6MWD estimation for the configuration with the best percentage error (i.e., “Watch + Shoes”). These plots aggregate the estimations of all 20 subjects in the cross-validation. In Appendix A, in Figure A13, Figure A14, Figure A15, Figure A16, Figure A17, Figure A18, Figure A19, Figure A20, Figure A21, Figure A22, Figure A23 and Figure A24, we report the correlation and Bland–Altman plots for the combinations, “Phone”, “Watch”, “Shoes”, “Phone + Watch”, “Phone + Shoes”, and “All Devices”.

Table 4 reports the GS and 6MWD estimation performances in terms of estimation errors (RMSE for GS and ε_r_ for 6MWD) for different sizes of the analysis window and for different device combinations. In general, the trend of GS estimation suggests an inverse relationship between the analysis window size and the estimation error, whereas an increase in the window size corresponds to a decrease in estimation error. However, the trend showed one exception. For the “phone” combination, increasing the window size from 5 to 10 s resulted in an increase in the estimation error of the GS. Regarding the estimation of the 6MWD, the trend was less defined, without a clear pattern between the estimation error and the analysis window size.

### 3.2. Feature Interpretability

The selection process for the most significant features, following the steps described in section E of the methods, yielded the following results:

1. Significant features: starting from 518 (148 from phones, 148 from watches, 222 from shoes), we obtained 123 features;
2. First drop-out stage: from 123, we dropped to 32;
3. Second drop-out stage: out of the thirty-two, only six were retained.

We outlined the features that emerged for every device. The mean ± std was computed considering the occurrences that successfully passed through the selection process:

A. Smartphone:
  - The Shannon entropy of the modulus of the accelerometer when low-pass filtered (β = 0.0527 ± 0.0162, tStat = 45.57 ± 12.57);
  - The mean crossing rate of the modulus of the orientation sensor when low-pass filtered (β = 0.0279 ± 0.0048, tStat = 36.70 ± 7.37);
  - The range of the y (vertical) component of the accelerometer when low-pass filtered (β = −0.1469 ± 0.0234, tStat = −54.20 ± 11.21);
  - The standard deviation of the y (vertical) component of the accelerometer when low-pass filtered (β = 0.255 ± 0.1159, tStat = 77.38 ± 40.48).
B. Smartwatch:
  - The root mean square of the modulus of the accelerometer when low-pass filtered (β = −0.279 ± 0.4715, tStat = 29.19 ± 47.73).
C. Smart shoes:
  - The mean of the z (vertical) component of the accelerometer of the left shoe when low-pass filtered (β = −0.0487 ± 0.0163, tStat = −39.58 ± 11.81).

Table 5 summarizes the features selected by learning the models of all 20 subjects. The feature names reported in the table are constructed in this way: “STATISTIC”_”SENSOR”_”FILTER MODE”_”COMPONENT”_”DEVICE”. STATISTIC: ENT->Shannon Entropy, RNG->Range, STD->Standard Deviation, MCR->Mean Crossing Rate, PF->Peak Frequency, SMA->Signal Magnitude Area, CV->Coefficient of Variation, MAX->max value, MEAN->mean value, RMS->Root Mean Square; SENSOR: acc->accelerometer, gyr->gyroscope, ori->orientation sensor, press->pressure sensor; FILTER MOD: lp->low-pass filtered, lp-hp->band-pass filtered; COMPONENT: x->*x* axis, y->*y*-axis, z->*z*-axis, mod->modulus; DEVICE: dev_1->phone, dev_2->watch, dev_3->left shoe, dev_4->right shoe.

## 4. Discussion

The results indicated that the models had reasonably accurate performance in estimating walking speed (mean RMSE: 0.119 ± 0.0132 m/s; minimum RMSE of 0.109 ± 0.029 m/s in the “watch + shoes” combination; maximum RMSE of 0.141 ± 0.05 m/s for the “watch”). We also used the extracted gait speed to estimate the 6MWD, i.e., the distance traveled in each six-minute walking trial. For the estimation of the 6MWD, we obtained a mean percentage error of 7.9 ± 0.9. We obtained the minimum percentage error of 6.6 ± 5.1 in the “watch + shoes” combination and the maximum percentage error of 9.3 ± 7.5 in the “shoes” combination.

Our results on GS estimation appear to be aligned with the previous literature, which, however, focused on methods applied to single device combinations. Soltani and Aminian [11], using a single inertial sensor worn on the lower back, obtained the best RMSE of 0.1 m/s across different contexts. Shresta and Won [12] used a smartphone placed in the trouser pocket and achieved an RMSE of 0.16 m/s, while McGinnis and colleagues [14] used three identical accelerometers placed on the sacrum, thigh, and shank, reaching a mean RMSE of 0.136 m/s. Interestingly, Soltani and colleagues [10], using only a wrist sensor, obtained an RMSE of 0.1 m/s, which improved up to 0.05 m/s when employing a personalized approach that took into account subject-specific gait characteristics obtained through a calibration phase utilizing the global navigation satellite system.

We observed that each single wearable device exhibited good performance in predicting gait speed, with small differences among them. Notably, the standalone smartphone outperformed other devices in GS prediction. However, the smartphone was consistently and artificially positioned in a controlled manner on the body. This represents a limitation, as this scenario is not always replicable in real life. Moreover, the controlled placement may influence the smartphone’s superior performance and raise questions about the generalizability of these findings to real living conditions, where device placement may vary.

The smartwatch showed a worse ability to predict GS compared to the smartphone, even if it presented a good accuracy of distance estimation with a very low bias. However, the smartwatch came with important advantages. Indeed, it has less intrinsic variability in wear placement across individuals, suggesting a better generalizability of the results. Moreover, when the smartwatch was paired with other devices, a good improvement in accuracy was observed, leading to the highest mean performance among all combinations. This synergistic effect suggests the potential for integrating multiple wearables to enhance overall predictive capabilities. It was worthwhile noting that the smartwatch also captures additional physiological information, such as heart rate (HR), that could potentially further enhance prediction accuracy. Indeed, as suggested by the work from Schubert et al. [25], HR-based features are predictors of 6MWD outcomes.

Quite surprisingly, wearing all devices simultaneously did not yield optimal performance. This counterintuitive result may be due to the redundancy in features extracted from different devices, leading to a complex and potentially conflicting input for the prediction models. The intricacies of combining data from different sensors may introduce noise and hinder the model’s ability to discern meaningful patterns, thereby decreasing predictive accuracy.

The analysis of the “best” features identified at the end of the validation (see section A.1 in III. Results section) highlights a noteworthy observation: the feature exerting the most significant influence on walking speed was the “root mean square of the modulus of the accelerometer when low-pass filtered”. This parameter exhibits considerable variability among subjects, indicative of the diverse walking styles within the study cohort. Notably, three of the six selected features pertained to the vertical component, underscoring the rich information vertical movement offers regarding walking speed. Additionally, it was intriguing to observe that five of the six identified features derived from accelerometer data, with the remaining feature sourced from the phone’s orientation sensor. This underscores the prominence of accelerometer-derived metrics in gauging walking speed.

Considering all combinations of wearable devices, it was possible to estimate GS with performances comparable to those in the literature (on average, the RMSE = 0.119 ± 0.0132 m/s, calculated with time windows of 5 s, considering all paces together) [10,11,12,14,26,27]. Moreover, the RMSE average seemed to improve with the size of time windows; the larger the size, the lower the RMSE. In fact, the performance, considering all paces together, changed from an average RMSE of 0.148 ± 0.02 m/s with a 2.5 s window to an RMSE of 0.1 ± 0.01 m/s with a 20 s window. Taking into account all the paces together, the best-performing device considered individually, as we expected, was the smartphone (RMSE = 0.114 ± 0.055), since it was placed in the trouser pocket and was the closest to the center of mass, whose speed was used as the reference system. If the devices were used in combination, the performance improved compared to the three individual devices, except for the combination “Phone + Watch”. It is interesting to note that “Watch” and “Shoes” taken singularly performed much worse than their combination, “Watch + Shoes”, which emerged as the overall best among all seven available (RMSE = 0.109 ± 0.029).

Our findings indicate that using multiple devices together could result in the reduced performance of GS estimation, possibly due to redundant features and complexity of the model. To improve accuracy and minimize the impact of redundant data, a feasible approach could be to employ dimensionality reduction techniques. Additionally, an alternative strategy could involve implementing a weighted Model Ensemble approach, which combines predictions from different devices used as separate and independent data sources. In this scenario, weights could be assigned to predictions derived from individual devices based on their relevance, applicability, and robustness.

The other parameter, the estimation of the 6MWD, which is derived from GS, was stable in all combinations of wearable devices in the trials at different paces with an average percentage error of 7.9 ± 0.9%. The best combination of devices considering the three paces together, even in this case, was the one that combined watch and shoes (ε_r_ = 6.6 ± 5.1). It is important to note that, in all the seven combinations available, the standard deviations were very wide, showing considerable intra-subject variability in the 6MWD estimation.

The results we obtained highlight that it is possible to estimate mobility parameters, such as gait speed or walking distance, with good accuracy using various combinations of wearable devices without any effort required from the user. The flexibility to use a standalone device or any combination tailored to the subject’s preferences has the potential to increase patient acceptance and enable the continuous daily life collection of mobility data. Indeed, this approach allows for greater compliance, as patients are not forced to use the same device all the time but can choose the one most suitable for their daily activities. This capability potentially enables day-by-day estimation of mobility parameters and their changes over time. For the COPD population, this implies a proactive assessment of physical capacity, leading to timely indications of worsening or improving conditions [28,29,30]. This supports the personalization of treatment plans and early intervention in case of sudden worsening, with the potential to reduce acute complications and improve the quality of life. Similar consideration could be used for other pathological conditions [31,32,33].

## 5. Conclusions

In conclusion, our study highlights the robust performance of various wearable devices, including smartphones, smartwatches, and smart shoes, in accurately estimating gait speed and distance traveled, which are crucial metrics in assessing mobility, especially in clinical contexts such as COPD. While individual devices exhibit good predictive capabilities, our findings highlight the potential for enhanced accuracy and generalizability when these devices are strategically combined. Notably, the smartwatch demonstrates promising capabilities, particularly when paired with other devices. Our results also shed light on the complex interplay between data fusion and predictive performance, with simultaneous device usage demonstrating diminishing returns likely due to feature redundancy and increased noise. These results suggest the importance of thoughtful integration strategies to maximize predictive accuracy while minimizing complexity. The implications of our findings extend beyond clinical settings to broader applications in personalized healthcare and remote monitoring. Indeed, combining different devices tailored to specific situations or patient preferences while maintaining good estimation performance enhances the potential for continuous monitoring and estimation of mobility parameters in daily life. This approach holds significant promise for enhancing the management and treatment of chronic diseases like COPD. It enables the continuous, non-invasive monitoring of a patient’s mobility and activity levels, considering the different needs of patients who may prefer one device over another based on the moment of the day and their health conditions.

## Figures and Tables

**Figure 4:**
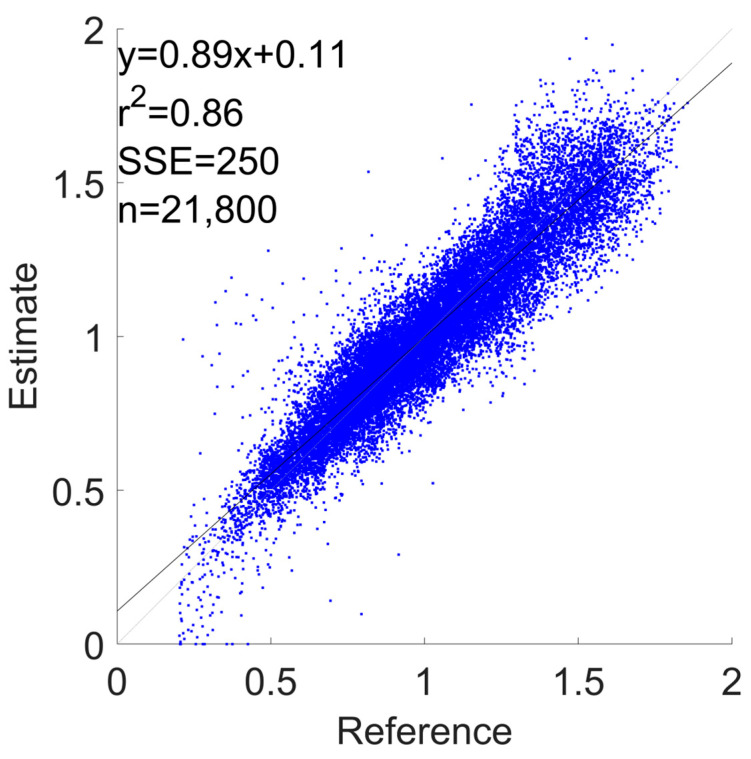
GS estimation using a correlation plot for the “Watch + Shoes” combination.

**Figure 5:**
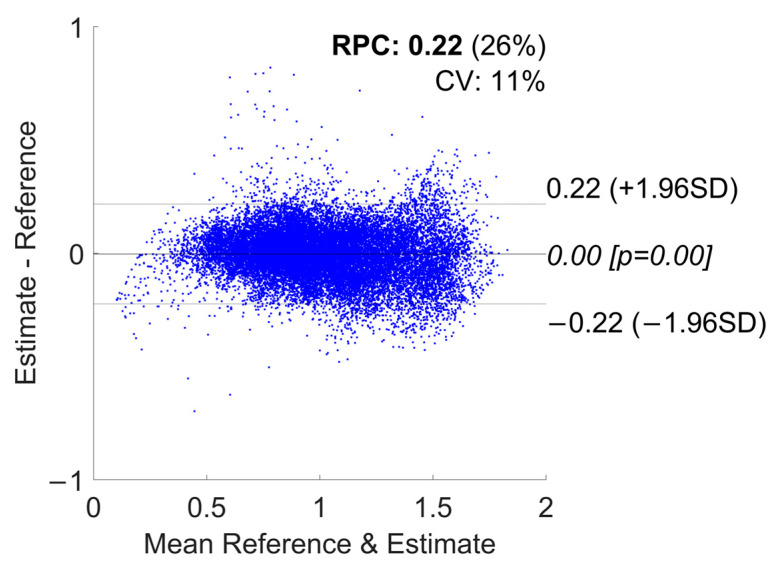
GS estimation using a Bland–Altman plot for the “Watch + Shoes” combination.

**Table 1:** Signals acquired per device.

Sensor	Units	Sampling Frequency (Hz)	Phone	Watch	Shoes
Accelerometer	m/s^2^	50	X, Y, Z	X, Y, Z	X, Y, Z
Gyroscope	rad/s	50	-	X, Y, Z	X, Y, Z
Orientation	deg	50	Azimuth-Pitch-Roll	-	-
Pressure	mV	50	-	-	X, Y, Z

**Table 2:** RMSE and Bland–Altman indices for GS estimation across different device combinations.

Devices Combination	RMSE [m/s]	Lower Limit of Agreement [−1.96 SD]	Upper Limit of Agreement [+1.96 SD]	Bias	R2
Phone	0.114 ± 0.055	−0.24	0.25	0.01	0.83
Watch	0.135 ± 0.033	−0.27	0.27	0.00	0.79
Shoes	0.141 ± 0.05	−0.3	0.29	−0.01	0.76
Phone + Watch	0.113 ± 0.051	−0.25	0.24	−0.00	0.84
Phone + Shoes	0.11 ± 0.04	−0.23	0.23	0.00	0.85
Watch + Shoes	0.109 ± 0.029	−0.22	0.22	0.00	0.86
All Devices	0.111 ± 0.043	−0.24	0.23	−0.00	0.85

**Table 3:** ε_r_ and Bland–Altman’s indices for the 6MWD estimation for different device combinations.

Devices Combination	ε_r_	Lower Limit of Agreement [−1.96SD]	Upper Limit of Agreement [+1.96SD]	Bias	R2
Phone	7.2 ± 7.1	−72	75	1.6	0.85
Watch	8.6 ± 6.7	−75	75	0.24	0.85
Shoes	9.3 ± 8.1	−92	86	−2.7	0.78
Phone + Watch	7.8 ± 6.6	−73	70	−1.6	0.86
Phone + Shoes	7.6 ± 5.5	−69	68	−0.72	0.87
Watch + Shoes	6.6 ± 5.1	−63	59	−1.7	0.9
All Devices	8.4 ± 6	−77	74	−1.5	0.85

**Table 4:** GS and 6MWD performance by varying the analysis window size and device combinations.

	2.5 s		5 s		10 s		20 s	
Devices Combination	GS (RMSE [m/s])	6MWD (ε_r_)	GS (RMSE [m/s])	6MWD (ε_r_)	GS (RMSE [m/s])	6MWD (ε_r_)	GS (RMSE [m/s])	6MWD (ε_r_)
Phone	0.145 ± 0.053	8 ± 7.6	0.114 ± 0.055	7.2 ± 7.1	0.108 ± 0.06	8.2 ± 7.1	0.101 ± 0.064	8.6 ± 6.7
Watch	0.179 ± 0.042	9.3 ± 8.2	0.135 ± 0.033	8.6 ± 6.7	0.122 ± 0.036	9.4 ± 7.0	0.116 ± 0.036	9.1 ± 6.5
Shoes	0.17 ± 0.038	9.6 ± 6.7	0.141 ± 0.05	9.3 ± 8.1	0.112 ± 0.047	7.5 ± 6.5	0.112 ± 0.05	8.5 ± 6.7
Phone + Watch	0.135 ± 0.038	7.2 ± 6.1	0.113 ± 0.051	7.8 ± 6.6	0.109 ± 0.058	8.5 ± 7	0.094 ± 0.051	7.6 ± 5.9
Phone + Shoes	0.134 ± 0.043	8.3 ± 6.5	0.11 ± 0.04	7.6 ± 5.5	0.095 ± 0.046	7.2 ± 5.1	0.1 ± 0.044	8.5 ± 6.2
Watch + Shoes	0.152 ± 0.035	8.5 ± 6.7	0.109 ± 0.029	6.6 ± 5.1	0.093 ± 0.041	6.4 ± 5.4	0.091 ± 0.043	6.9 ± 5.8
All Devices	0.124 ± 0.031	7.9 ± 5.4	0.111 ± 0.043	8.4 ± 6	0.098 ± 0.036	7.8 ± 4.9	0.088 ± 0.041	7.2 ± 6

**Table 5:** Estimate ± SE, (all *p*-values < 0.001).

	Phone	Watch	Shoes	Phone + Watch	Phone + Shoes	Watch + Shoes	All Devices
Intercept	0.951 +/− 0.001	0.945 +/− 0.001	0.971 +/− 0.001	0.954 +/− 0.001	1.375 +/− 0.011	0.963 +/− 0.001	0.955 +/− 0.001
Feat. 1	ENT_acc_lp_mod_dev_1: 0.062 +/− 0.001	RNG_acc_lp_mod_dev_2: −0.114 +/− 0.004	MEAN_acc_lp_mod_dev_3: 0.14 +/− 0.002	STD_acc_lp_mod_dev_1: 0.373 +/− 0.006	ENT_acc_lp_mod_dev_1: 0.032 +/− 0.001	RMS_acc_lp_mod_dev_2: 0.079 +/− 0.001	MCR_acc_lp_mod_dev_1: −0.03 +/− 0.001
Feat. 2	RNG_acc_lp_y_dev_1: −0.143 +/− 0.003	RMS_acc_lp_mod_dev_2: −0.9 +/− 0.028	MEAN_acc_lp_z_dev_3: −0.02 +/− 0.002	MCR_acc_lp_mod_dev_1: −0.035 +/− 0.001	RNG_acc_lp_y_dev_1: −0.122 +/− 0.003	STD_acc_lp_mod_dev_2: 0.086 +/− 0.001	ENT_acc_lp_mod_dev_1: 0.03 +/− 0.001
Feat. 3	MEAN_acc_lp_y_dev_1: −0.021 +/− 0.001	MEAN_acc_lp_mod_dev_2: 0.908 +/− 0.025	ENT_acc_lp-hp_y_dev_3: 0.048 +/− 0.001	ENT_acc_lp_mod_dev_1: 0.065 +/− 0.001	STD_acc_lp_y_dev_1: 0.291 +/− 0.003	ENT_acc_lp_mod_dev_2: 0.037 +/− 0.001	RNG_acc_lp_y_dev_1: −0.128 +/− 0.003
Feat. 4	STD_acc_lp_y_dev_1: 0.374 +/− 0.004	STD_acc_lp_mod_dev_2: 0.437 +/− 0.008	MCR_pre_lp_mod_dev_3: 0.043 +/− 0.001	MAX_acc_lp_y_dev_1: −0.09 +/− 0.002	MCR_gyr_lp_x_dev_1: 0.031 +/− 0.001	CV_gyr_lp-hp_mod_dev_2: 0.025 +/− 0.001	STD_acc_lp_y_dev_1: 0.292 +/− 0.003
Feat. 5	MCR_acc_lp_y_dev_1: −0.035 +/− 0.001	ENT_acc_lp_mod_dev_2: 0.028 +/− 0.002	PF_pre_lp_z_dev_3: 0.053 +/− 0.001	STD_acc_lp_y_dev_1: 0.019 +/− 0.006	PF_gyr_lp_y_dev_1: 0.022 +/− 0.001	MEAN_acc_lp_z_dev_3: −0.063 +/− 0.001	MCR_gyr_lp_mod_dev_1: 0.03 +/− 0.001
Feat. 6	MCR_acc_lp-hp_mod_dev_1: 0.039 +/− 0.001	MEAN_acc_lp_y_dev_2: 0.028 +/− 0.001	MAX_pre_lp-hp_x_dev_3: 0.032 +/− 0.001	PF_acc_lp_z_dev_1: 0.028 +/− 0.001	PF_gyr_lp-hp_z_dev_1: 0.019 +/− 0.001	ENT_gyr_lp_z_dev_3: 0.044 +/− 0.001	SMA_gyr_lp-hp_dev_2: 0.058 +/− 0.001
Feat. 7	PF_acc_lp-hp_mod_dev_1: 0.02 +/− 0.001	SMA_acc_lp-hp_dev_2: 0.054 +/− 0.003	ENT_acc_lp_x_dev_4: 0.025 +/− 0.001	SMA_acc_lp-hp_dev_1: 0.068 +/− 0.003	MEAN_acc_lp_z_dev_3: −0.047 +/− 0.001	RMS_acc_lp_z_dev_4: 0.071 +/− 0.00	MEAN_acc_lp_z_dev_3: −0.045 +/− 0.001
Feat. 8	PF_acc_lp-hp_z_dev_1: 0.028 +/− 0.001	MCR_acc_lp-hp_mod_dev_2: 0.051 +/− 0.002	RMS_acc_lp_z_dev_4: 0.117 +/− 0.002	STD_acc_lp-hp_mod_dev_1: −0.165 +/− 0.004	CV_gyr_lp_mod_dev_3: 1.922 +/− 0.051	RMS_gyr_lp_z_dev_4: −0.047 +/− 0.001	MEAN_acc_lp_z_dev_4: −0.045 +/− 0.001
Feat. 9	MCR_gyr_lp_mod_dev_1: 0.025 +/− 0.001	CV_gyr_lp_mod_dev_2: 0.039 +/− 0.001	MCR_gyr_lp-hp_z_dev_4: 0.041 +/− 0.001	MCR_gyr_lp_mod_dev_1: 0.032 +/− 0.001	MAX_pre_lp_z_dev_3: 0.023 +/− 0.001	MEAN_gyr_lp-hp_mod_dev_4: 0.05 +/− 0.002	RMS_gyr_lp_x_dev_4: 0.032 +/− 0.001
Feat. 10	PF_gyr_lp-hp_z_dev_1: 0.024 +/− 0.001	RNG_gyr_lp_y_dev_2: −0.046 +/− 0.002	ENT_pre_lp-hp_y_dev_4: 0.036 +/− 0.001	RMS_acc_lp_mod_dev_2: 0.074 +/− 0.001	MEAN_acc_lp_z_dev_4: −0.069 +/− 0.001	ENT_pre_lp_z_dev_4: 0.031 +/− 0.001	ENT_gyr_lp-hp_x_dev_4: 0.032 +/− 0.001

**Figure A1:**
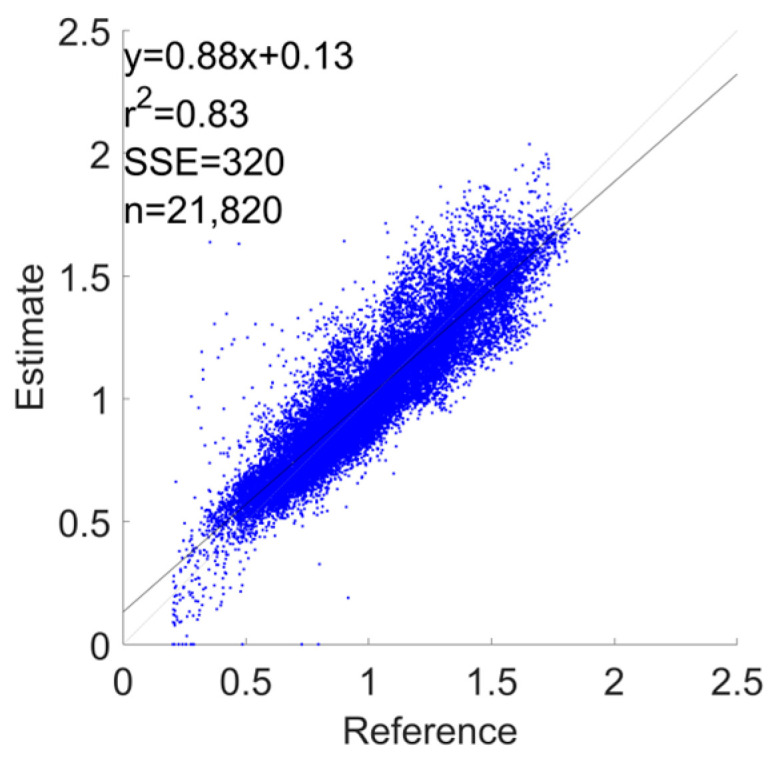
GS estimation using a correlation plot for “Phone” configuration.

**Figure A2:**
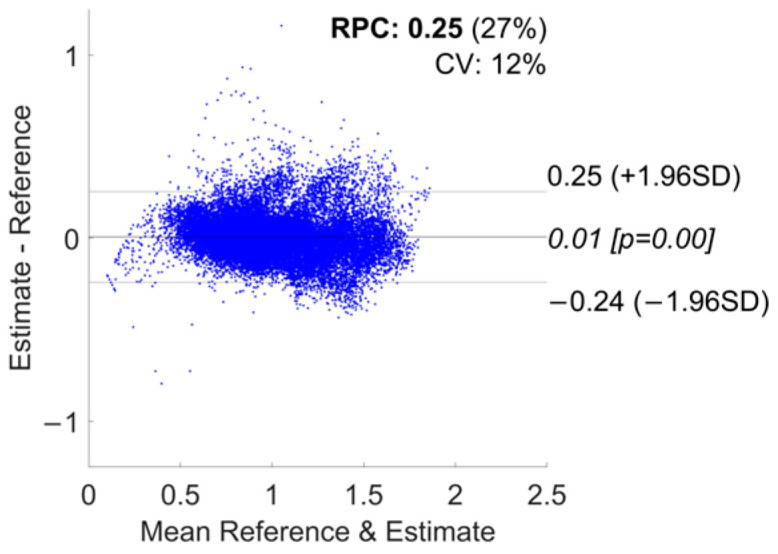
GS estimation using a Bland–Altman plot for “Phone” configuration.

**Figure A3:**
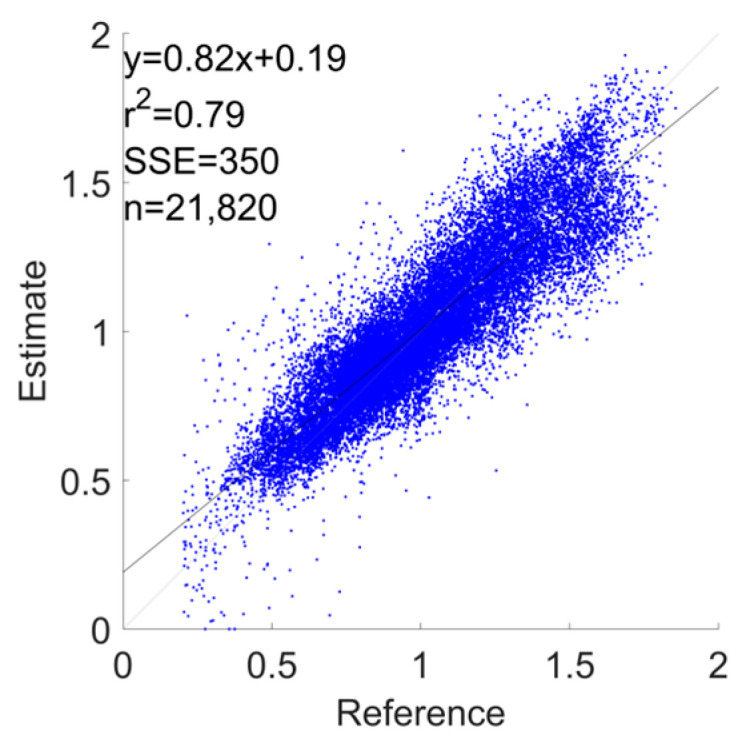
GS estimation using a correlation plot for “Watch” configuration.

**Figure A4:**
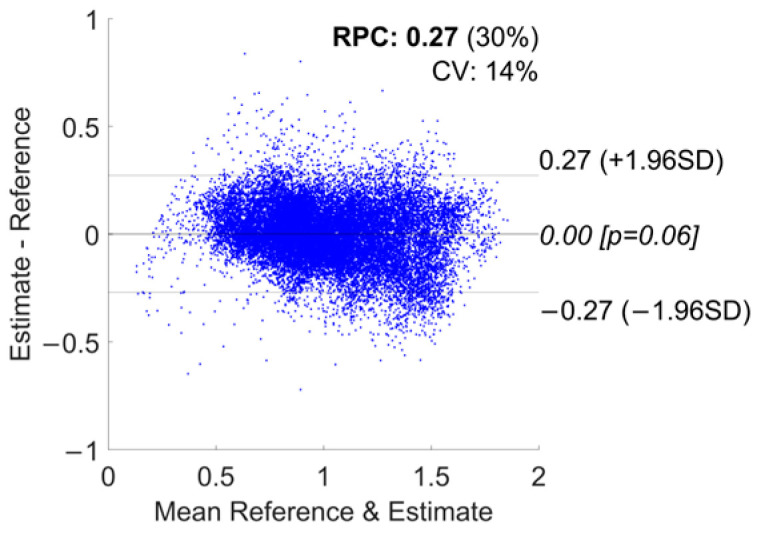
GS estimation using a Bland-Altman plot for “Watch” configuration.

**Figure A5:**
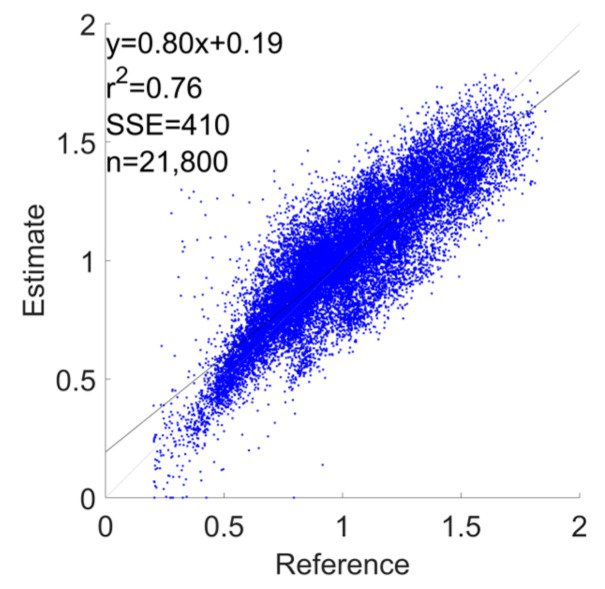
GS estimation using a correlation plot for “Shoes” configuration.

**Figure A6:**
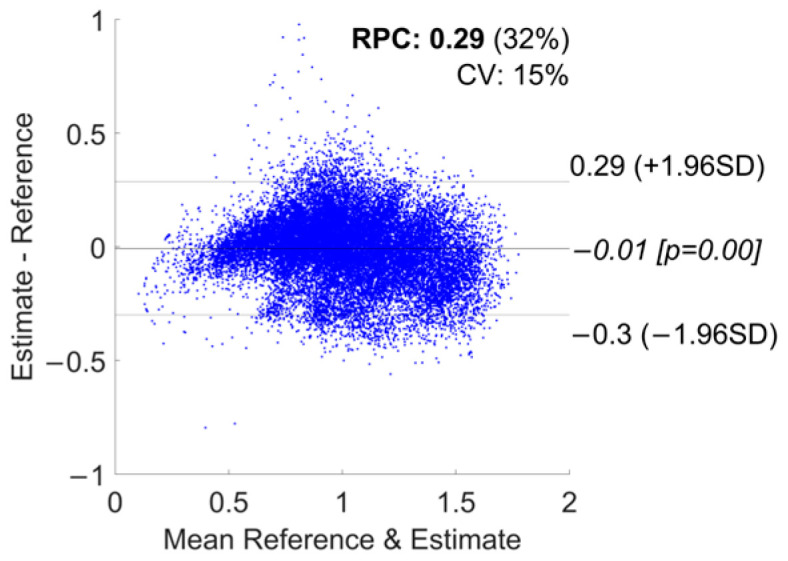
GS estimation using a Bland–Altman plot for “Shoes” configuration.

**Figure A7:**
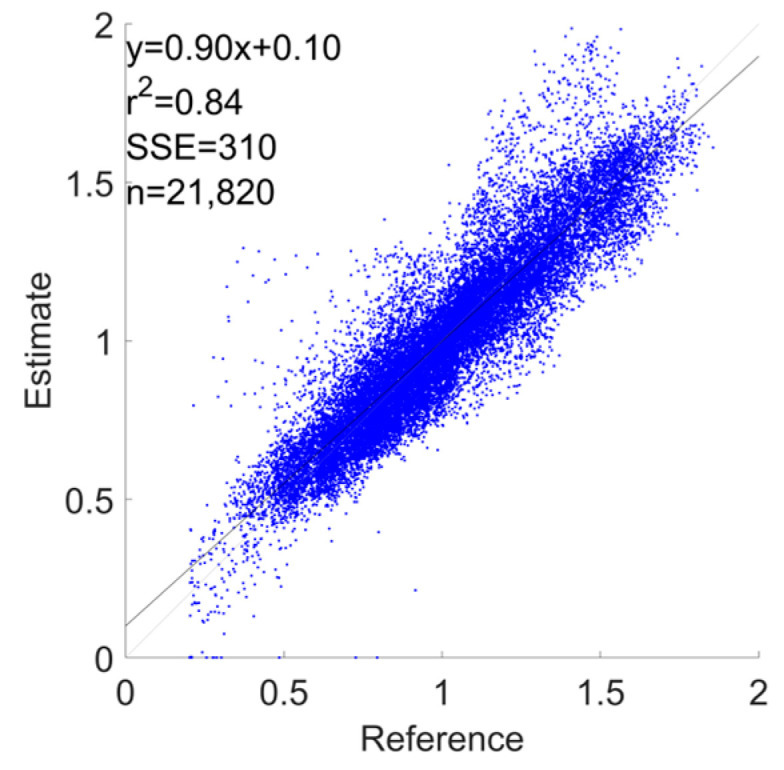
GS estimation using a correlation plot for the “Phone + Watch” configuration.

**Figure A8:**
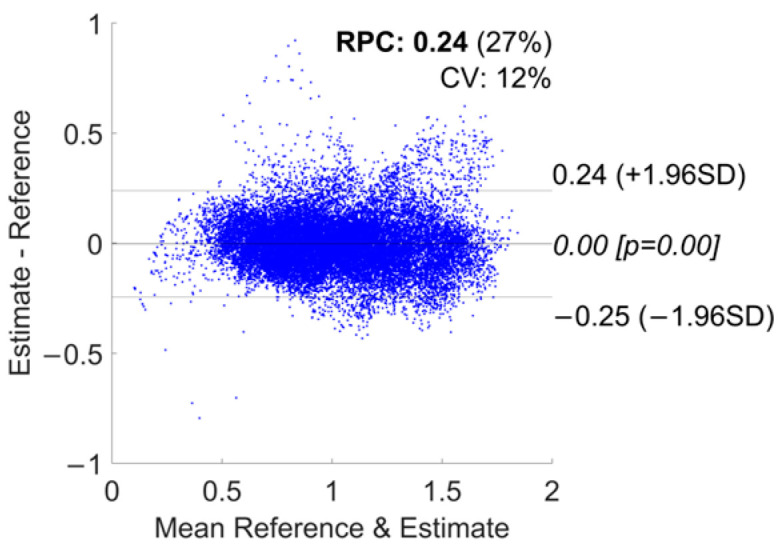
GS estimation using a Bland–Altman plot for “Phone + Watch” configuration.

**Figure A9:**
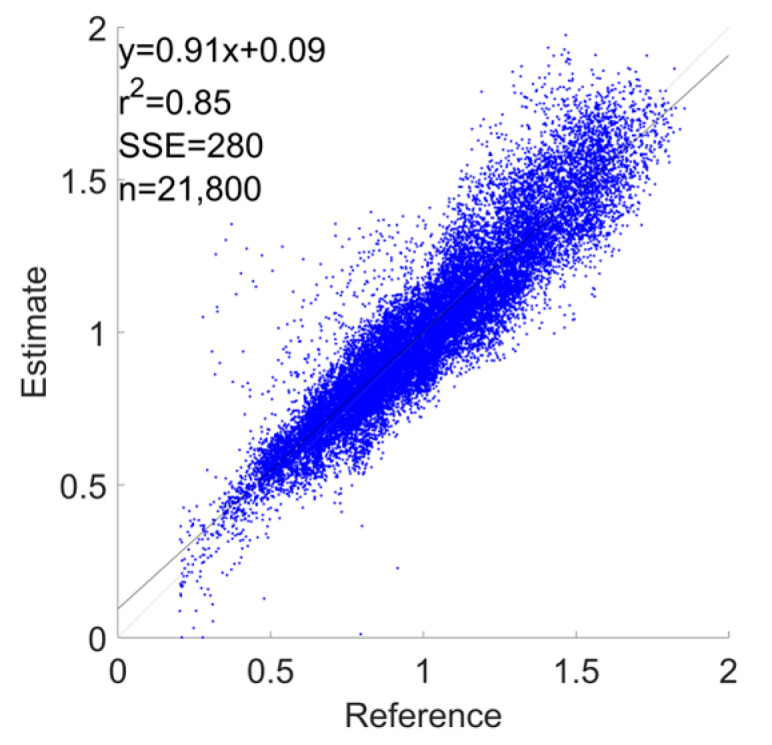
GS estimation using a correlation plot for the “Phone + Shoes” configuration.

**Figure A10:**
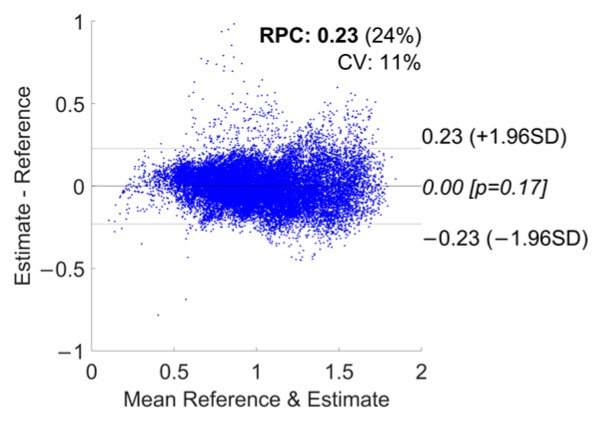
GS estimation using a Bland–Altman plot for “Phone + Shoes” configuration.

**Figure A11:**
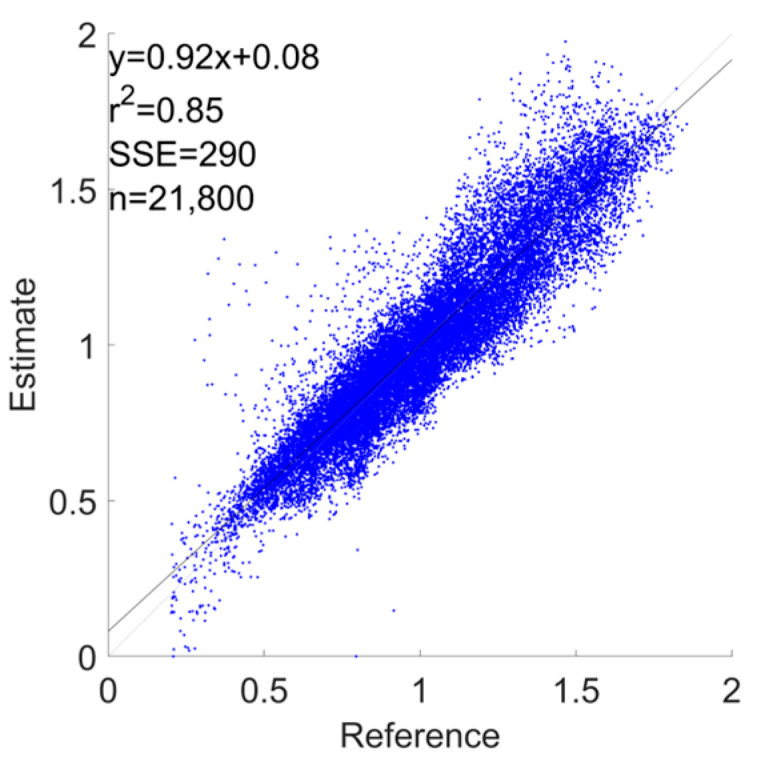
GS estimation using a correlation plot for “All Devices” configuration.

**Figure A12:**
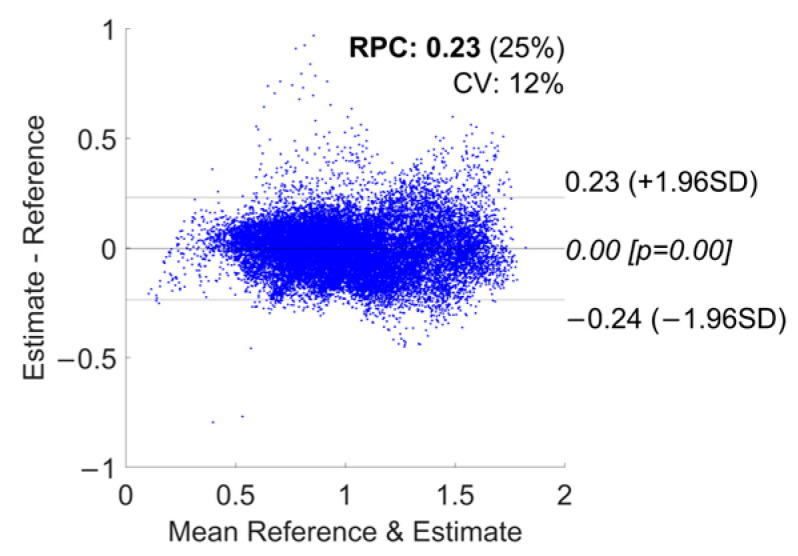
GS estimation using a Bland–Altman plot for “All Devices” configuration.

## Data Availability

The dataset is available on Zenodo (https://doi.org/10.5281/zenodo.11091279, accessed on 29 April 2024).
